# Design of bionic porous structure of bone scaffolds and analysis of fatigue and permeability characteristics

**DOI:** 10.3389/fbioe.2026.1765370

**Published:** 2026-03-04

**Authors:** Long Chao, Qun Chao, Qiuyuan Feng, Jianwei Guo, Wei Zhang, Yueqin Zhang, Jie Cui, Xing Chen, Ming Zhang

**Affiliations:** 1 School of Intelligent Manufacturing, Nanjing Vocational College of Information Technology, Nanjing, China; 2 Jinling Hospital, Affiliated Hospital of Medical School, Nanjing University, Nanjing, China; 3 Department of Hematology, Zibo Central Hospital, Zibo, China; 4 College of Mechanical and Electrical Engineering, Nanjing University of Aeronautics and Astronautics, Nanjing, China; 5 School of Mechanical Engineering, Suzhou University of Science and Technology, Suzhou, China

**Keywords:** bionic porous structures, bone implant, fatigue, permeability, voronoi diagram

## Abstract

Bone defects caused by trauma, malignant tumors, and other diseases are common symptoms in orthopedic surgery, and when they exceed the critical size of autologous repair, bone transplantation is required. Artificial bone scaffolds are an effective way to repair large bone defects. An ideal artificial bone scaffold should not only have appropriate mechanical properties and biocompatibility, but also have good osteoinductive properties. This paper proposed a bionic porous structure design method based on Voronoi diagram. In the design process, controlling pore morphology and pore size by adjusting irregularity and porosity. In addition, the designed scaffolds were fabricated through laser powder bed fusion (L-PBF) with Ti-6Al-4V powders. The mechanical characteristics were evaluated by static mechanical simulation with ABAQUS and fatigue tests, and the permeability characteristics were analyzed by fluid simulation with COMSOL and *in vitro* test. The research results showed that fatigue meet the natural bone implant criteria by controlling the porosity and irregularity. And the mathematical relationship between fatigue characteristics and porosity and irregularity parameters was fitted. It can effectively predict the fatigue characteristics of the scaffold and improve its application in natural bone. At the same time, the designed structure has closer permeability to the natural bone, and was conducive to cell proliferation. Therefore, the porous structure based on Voronoi diagram may have good application potential in the bionic design of bone implants.

## 1 Introduction

At present, autologous bone transplantation, artificial prosthesis and allograft are mainly used in the clinical treatment of bone defects and bone repair. However, the limited source of autologous bone, the disease transmission of allograft bone and the high elastic modulus of artificial prosthesis limit its clinical application. In clinical practice, the leading causes of severe bone defects include severe trauma, tumor resection or knee joint repair, which often require the replacement of the bone with an external implant (Ngo et al., 2018; Shao et al., 2017; Lu et al., 2019). However, the traditional external implants are limited by a mismatch between elastic moduli of the implant and the human bone, resulting in an inability to transfer stress from the implant to the human bone (Ghorbani et al., 2020; Akpan et al., 2020; Moreno et al., 2019; Zhao et al., 2019). Consequently, the interface between the implant and the human bone becomes loose, eventually leading to implant failure. In order to solve this problem, many studies have studied the non-solid bone implant structure with certain porosity and controlled the porosity through parameters, which is called porous structure (Rodríguez-Montaño et al., 2018; Yang et al., 2019; Chiu et al., 2019). The elastic modulus was changed to address the problem of “stress shielding” by adjusting the structural morphology. In addition, the structure with certain pores can provide good physical conditions for nutrition transmission, tissue growth and bone combination after bone implantation. With the development of 3D printing technology, the defect that traditional methods cannot prepare complex structures has been overcome, and the rapid and accurate manufacturing has been realized. Among them, metal materials are widely used in 3D printing of bone implants, and titanium alloy is relatively common, which has high mechanical strength and excellent biocompatibility, promoting the application of metal bone implants in medical engineering (Weißmann et al., 2016; Chang et al., 2020; Wang et al., 2019; Falkowska et al., 2020).

In terms of pore shape, porous structure can be divided into regular and irregular. Regular porous structure is composed of regular shape unit cell array, mainly including cube structure, rhombus structure or rhombus dodecahedron, with elastic moduli 0.5–15 GPa, 1.3–1.9 GPa, and 0.5–6.5 GPa, and strength limits of 20–200 MPa, 28.5–30 MPa, and 10–100 MPa, respectively (Rodríguez-Montaño et al., 2018; Wang et al., 2020; Wettergreen et al., 2005; Cai et al., 2019), at the same time, it has poor fatigue characteristics. The design method of irregular porous structure is different from regular porous structure, which is usually directly formed. Previous studies involving irregular porous structures often focused on gradient changes or changes in the shape of different pore-struts, without investigating changes in aperture density (Wang et al., 2019; Han et al., 2018; Wang Y. et al., 2018; Liu et al., 2018; Nune et al., 2017; Zhang X. et al., 2020). For example, the irregular porous structures studied only involved gradients (Wang G. et al., 2018; Du et al., 2020; Du et al., 2019; Liu et al., 2021), and mostly focused on quasi-static properties of the porous structures, however, the fatigue and permeability characteristic have yet to be studied in depth. The porous scaffold most common cause of fracture is fatigue failure, which may be low-cycle or high-cycle, the low-cycle fatigue failure occurs when the number of external load cycles is low, it is often accompanied by plastic deformation, the number of cycles is generally less than 10^4^. The high-cycle fatigue failure occurs when the number of external load cycles is high (Kelly et al., 2019; Zhao et al., 2018; Amin et al., 2013), indicates that the compressive strength is far beyond the stress. Under normal circumstances, and the number of cycles is generally higher than 10^4^ times (Amin et al., 2015).

Based on this, the design method of bionic porous structure using Voronoi principle is proposed in this paper, compared with the regular porous, the pore morphology and porosity can be controlled according to specific rules. The generated pore morphology is similar to human bone tissue, and the pore shape and size can be controlled by parameters, belongs to an irregular porous structure. Then analyze the effects of porosity and irregularity on fatigue and permeability characteristic, and the fatigue characteristics and design parameters were fitted by linear regression equation. The equation provided a reasonable estimate of the fatigue characteristic. Finally, the permeability characteristic was studied via fluid simulation and *in vitro* test, which provide theoretical guidance for the application of bone implants in medical engineering.

## 2 Materials and methods

### 2.1 Design method

**FIGURE 1:**
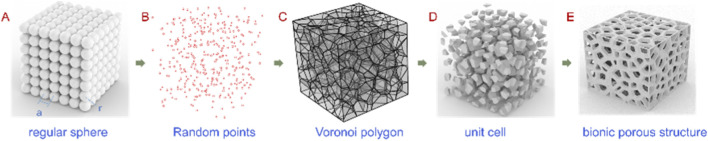
The bionic porous structure design process. **(A)** Regular spheres; **(B)** Random points; **(C)** Voronoi polygon; **(D)** Unit cell; **(E)** Bionic porous structure.

The proposed design method of bionic porous structure is based on Voronoi diagram. The specific design involves the following steps: (1) As shown in Figure 1A, a number of sphere matrices are arranged in the design area, called a probability sphere, the number of probability spheres determines the number of seed points; (2) As shown in Figure 1B, a random point in each sphere is selected as the seed point, and the density of seed points is determined by the radius (denoted as *r*) of the sphere and the spacing (denoted as *a*) between adjacent spheres; (3) As shown in Figure 1C, generate Voronoi polygons based on random points; (4) As shown in Figure 1D, each cell of Voronoi polygon is scaled, the smaller the cell, the smaller the pore size; (5) As shown in Figure 1E, extraction of the gap between each unit cell and smoothening to generate bionic porous structures.

**FIGURE 2:**
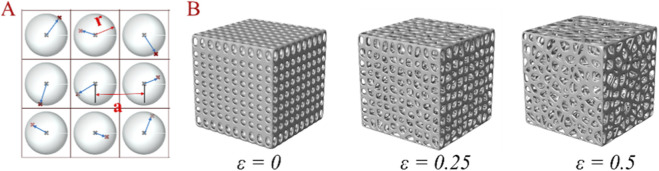
Schematic diagram of ε physical meaning and corresponding Voronoi scaffold models. **(A)** Seed point distribution principle (a: basic lattice spacing; r: random offset radius); **(B)** Voronoi scaffold models with different ε values (ε = 0: regular pores; ε = 0.25: moderately irregular pores; ε = 0.5: highly irregular pores).

The bionic porous structures should have different functionalities at different locations. This design method can control the fatigue and permeability characteristics of the structure by adjusting the pore morphology and porosity, while improving fatigue characteristics, and help promote nutrient transport and cell growth. However, the pore morphology is determined by the sphere radius *r* and spacing *a*. In order to better characterize the pore morphology, irregularity (*ε*) was introduced, which is the ratio of the *r* and *a*, that is, *ε* = *r*/*a*, the larger the irregularity, the more irregular the pore morphology, as shown in Figure 2.

In order to study the effects of bionic porous structures on the fatigue and permeability characteristics, we designed the porosity (*Φ*) into three different gradients of 75%, 82%, and 89% to match the physical properties of human bone. At each porosity, three groups different irregularity (*ε* = 0, 0.25, 0.5) were designed. Considering that the pore size of bone tissue is 200–1,200 μm (Hu et al., 2010; Wang et al., 2017; Lopes et al., 2018), so the point spacing is set to 1,500 μm, and 9 sets of design parameters are set as shown in Table 1.

**TABLE 1:** Design parameters of bionic porous structures.

*ε*	*Φ*	*a*	*r*
0	75%	1,500	0
	82%	1,500	0
	89%	1,500	0
0.25	75%	1,500	375
	82%	1,500	375
	89%	1,500	375
0.5	75%	1,500	750
	82%	1,500	750
	89%	1,500	750

### 2.2 Fatigue test

Before the fatigue test, we used L-PBF equipment (NCL-M2120, China) to prepare nine groups of Ti6Al4V scaffolds. The processing parameters are: laser power 140 W, hatch spacing 0.06 mm, scanning speed 1,200 mm/s, layer thickness 30 μm. The prepared scaffold was compressed on a mechanical testing machine, and then the accuracy of the prepared scaffold was analyzed by using an industrial CT machine (XTH 225, Nikon, Japan).

The quasi-static compression experiment was conducted using an electronic universal testing machine (CMT5105, MTS system, United States). According to the ISO 13314:2011 porous metal compression standard, the head speed is 2 mm/min. Extract the apparent elastic modulus and yield strength from the stress-strain curve. A finite element analysis (FEA) was conducted on the scaffold using commercial software ABAQUS (2016, Dassault System Simulia, United States), with standard material parameters of Ti6Al4V and applied pressure of 255 MPa. Evaluate the stress distribution of the scaffold under pressure through parameter setting, grid partitioning, and solution.

Dynamic high cycle fatigue testing was conducted using an electric hydraulic servo fatigue testing system (MTS809, MTS system, United States). The sine wave loading mode is set to 50 kN; the stress ratio is 0.2, this ratio simulates the dynamic load characteristics of bone, consistent with the stress ratio used in Ti-6Al-4V implant fatigue studies (Amin et al., 2015); and the frequency is 30 Hz, Human daily activities (e.g., walking) generate 1–10 Hz cyclic loads on bone (Kelly et al., 2019); 30 Hz is selected to balance test efficiency and physiological relevance, as recommended by ISO 13314:2011 for porous metal fatigue tests. It should be noted that the stress level is set based on the yield strength of the specimen. Using 17 μm industrial micro-CT scanner (FF35, YXLON International, Germany) scans samples to observe internal defects in fatigue experimental samples.

### 2.3 Permeability test

In order to analyze the permeability characteristics of bionic porous structure, the fluid simulation and cell experiment were used. Firstly, COMSOL software was used for fluid simulation analysis of the designed structure. In the process of fluid simulation, Reynolds number was used to analyze the flow state of the fluid; inlet velocity: 0.1 mm/s; outlet pressure: 0 MPa; wall boundary: no-slip condition; fluid density ρ=1000 kg/m^3^; dynamic viscosity μ=0.001 Pas; model length L=10 mm; mesh size: 20 μm. Meanwhile, since the physical condition of the simulation was an incompressible fluid with constant density, the following Navier-Stokes equation was used for analysis (Zhang et al., 2019; Ali and Sen, 2017):

$$\rho \frac{\partial u}{\partial t}-\mu \nabla^{2}u+\rho \left(u\cdot \nabla \right)u+\nabla p=F$$ (1)

where ρ indicates the density of the fluid (kg/m^3^); μ indicates the speed of the fluid (m/s); t indicates time (s); ∇ indicates the operator; p indicates pressure (MPa); u indicates the dynamic viscosity coefficient of the fluid; and F indicates the force (N).

In order to analyze the permeability and pressure drop of the bionic porous structure, Darcy’s law is cited in this paper to calculate it as follows (Zhao et al., 2016; Zhang L. et al., 2020):

$$K=v_{D}\cdot \mu_{d}\cdot \left(\frac{L}{\Delta P_{i-0}}\right)$$ (2)

$$\Delta P=P_{Inlet}-P_{Outlet}$$ (3)

Where K indicates the permeability; $v_{D}$ indicates Darcy velocity; μ _*d*_ indicates the dynamic velocity; L indicates the model length; and P indicates the pressure gradient of the fluid domain.

The MC3T3-E1 mouse cells were used for in cell test, which were commercially acquired for research use from the Cell Culture Center, Shanghai Institutes for Biological Sciences, Chinese Academy of Sciences (CAS). Before the cell test, put the scaffold in alcohol, wash it in ultrasonic cleaner for 30 min, soak it in distilled water for 10 min, then prepare 3M sodium hydroxide solution to remove the oxidation of the soaked scaffold, and finally clean the scaffold with distilled water and conduct high-temperature sterilization. In the cell test process, the cells were cultured with mouse osteoblasts in the medium at 37 °C and 5% CO_2_, and the medium was replaced every 2 days. In order to evaluate the cell activity, the cells were stained with fluorescence. After 4 days of culture, the results of cell staining were detected by confocal microscopy. In order to further observe the cell adhesion on the scaffold surface, after 7 days of cell culture, the scaffolds were fixed with 4% paraformaldehyde for 24 h, dehydrated through a gradient ethanol series (30%, 50%, 70%, 90%, 100%), and freeze-dried. To observe the inner surface of pores, the scaffolds were cut along the cross-section using a low-speed diamond saw (to avoid damaging cells, and the cell adhesion on the scaffold surface was observed by SEM.

## 3 Results and discussion

### 3.1 Fatigue characteristics

**FIGURE 3:**
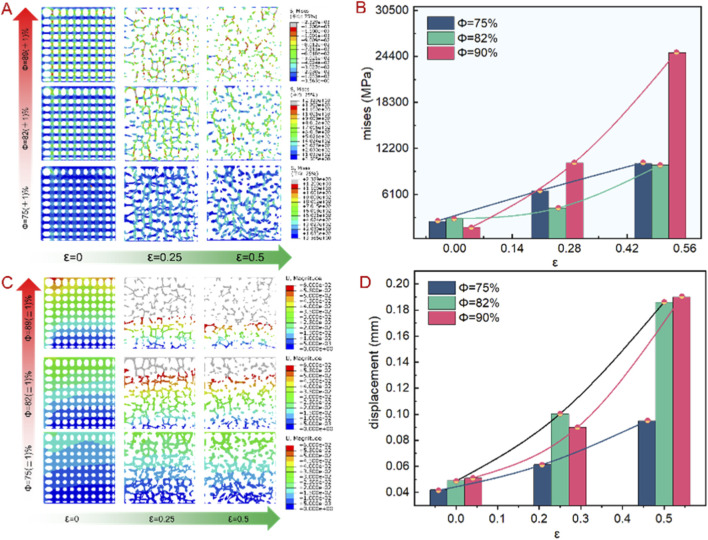
Mechanical finite element results of bionic porous structures. **(A)** Mise stress cloud diagram. **(B)** Mise stress value trend. **(C)** Displacement cloud diagram. **(D)** Displacement value trend.

Before the fatigue test, in order to select appropriate stress parameters for the test, it is necessary to conduct static compression test on the scaffold. Firstly, the Abaqus was used to mechanical finite element analysis. Figures 3A,B shows the stress change trend corresponding to different porosity and irregularity. It can be seen that under the same constraint condition, the stress concentration mainly occurs at the node and the thinner rod, and the stress spreads from the node to the surrounding, and the stress increases with the increased irregularity. Which indicates that the greater the irregularity, the lower the capacity to bear additional stress, the implanted bone can bear less external load. When the irregularity is greater than 0.25, the higher the porosity, the greater the stress, and the more likely to fracture. As shown in Figures 3C,D, under similar load, the displacement increases with the increase of the irregularity and porosity. When the irregularity was 0.5 and the porosity was 90%, the displacement was the largest, with a maximum value of 0.19. Thus, the greater the porosity and irregularity, the greater is the displacement, and lower is the elastic modulus. Therefore, in the design process of bionic porous structures, the porosity and irregularity are critical parameters. The prevention of structural fractures caused by controlling the porosity and the irregularity can effectively enhance its mechanical properties.

On the basis of mechanical finite element analysis, in order to ensure the effectiveness of the mechanical test, it is necessary to analyze the forming accuracy of the scaffold, compare the porosity from CT data and the designed model, and calculate its deviation. Table 2 shows that the deviation was basically within 3%. Also, the porosity of the actual printed sample was less than the porosity of the designed model, mainly due to the presence of incompletely melted powder during the printing process. Thus, the printed porous structure scaffold basically met the design requirements, and the L-PBF printing equipment exhibits high accuracy, it also demonstrates the scientific and rationale of the subsequent experiments.

**TABLE 2:** Porosity deviation of the design model and the printed sample.

Group	Design porosity	Actual porosity	Deviation
1 (*Φ* = 75%, *ε* = 0)	75.0%	72.8%	2.2%
2 (*Φ* = 75%, *ε* = 0.25)	75.0%	72.9%	2.1%
3 (*Φ* = 75%, *ε* = 0.5)	75.0%	72.1%	2.9%
4 (*Φ* = 82%, *ε* = 0)	82.0%	79.4%	2.6%
5 (*Φ* = 82%, *ε* = 0.25)	82.0%	79.1%	2.9%
6 (*Φ* = 82%, *ε* = 0.5)	82.0%	79.3%	2.7%
7 (*Φ* = 89%, *ε* = 0)	89.0%	86.2%	2.8%
8 (*Φ* = 89%, *ε* = 0.25)	89.0%	86.1%	2.9%
9 (*Φ* = 89%, *ε* = 0.5)	89.0%	86.3%	2.7%

**FIGURE 4:**
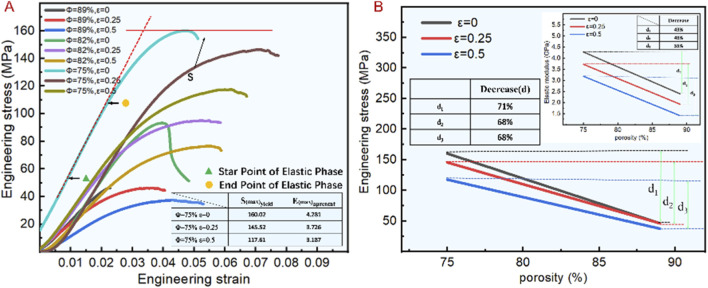
Compression experiment results of bionic porous scaffolds. **(A)** Stress–strain curve of different irregularities and porosities. **(B)** Compressive strength and elastic modulus with different porosities and irregularities.

The mechanical properties of bionic porous structure are characterized by analyzing its apparent modulus of elasticity (*E*) and compressive strength (*S*) (Amin et al., 2014; Li et al., 2020). Figure 4A shows the stress-strain curves corresponding to different design parameters, where the elastic modulus is the slope of the curve, and the compressive strength is the maximum value of the curve. When the porosity was 75%, the scaffold fracture occurred at maximum stress. As shown in Figure 4B, the compressive strength did not differ significantly when the irregularity was 0 and 0.25. However, when the irregularity was 0.5, it reached the lowest value, the elastic modulus ranged from 1.414 to 4.281 GPa as the irregularity increased, the elastic modulus decreased, the higher the porosity, the greater was the effect of irregularity on the elastic modulus.

As shown in Figure 4B, the greater the irregularity of the scaffold, the higher the corresponding elastic modulus and compressive strength. When the irregularity is 0, the compressive strength and elastic modulus decrease by 71% and 43% with the porosity increase. When the irregularity increases to 0.25, the compressive strength and elastic modulus decrease by 68% and 48% with the porosity increase. When the irregularity reaches the maximum value of 0.5, the compressive strength and elastic modulus decrease by 68% and 55%. It shows that the compressive strength is greatly affected by the porosity, the elastic modulus is relatively small, and under different porosity. The influence of irregularity on the compressive strength was little, from 68% to 71%, but the elastic modulus changes from 43% to 55%. Indicating that the influence of irregularity on the elastic modulus is greater than the compressive strength. So it is important to control the irregularity when solving the stress shielding problem of bone implants.

**FIGURE 5:**
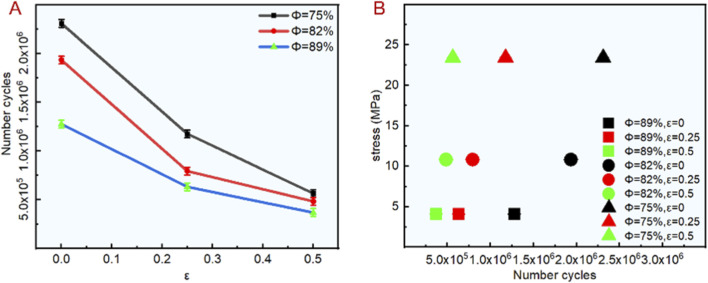
Fatigue test results of bionic porous structures. **(A)** Number of cycles. **(B)** Stress failure in different number of cycles.

Based on the compression experiment, the fatigue characteristics of the bionic porous structure are analyzed. We considered 25% of the compressive strength as the maximum compressive stress. *In vivo*, bone implants bear cyclic loads equivalent to 10%–30% of their compressive strength during daily activities (e.g., walking, standing) (Kelly et al., 2019; Amin et al., 2013); 25% is a widely adopted stress level for bone scaffold fatigue tests (Zhao et al., 2018). A sinusoidal loading method was adopted for the force loading. The minimum compressive stress in the nine curve was 10% of the maximum compressive stress, and the loading frequency was 10 Hz. Figure 5A shows the fatigue test results with different porosity and irregularities. Among these, the number of cycles with porosity of 75%, 82%, and 89% ranged from 56 × 10^4^ to 23 × 10^5^, 48 × 10^4^ to 19 × 10^5^ and 36 × 10^4^ to 12 × 10^5^ respectively. The number of cycles with an irregularity of 0.5, 0.25, 0 ranged from 36 × 10^4^ to 56 × 10^4^, and 63 × 10^4^ to 11 × 10^5^, 12 × 10^5^ to 23 × 10^5^. The test results showed that the irregularity had a great influence on the fatigue characteristic. As the irregularity increased, the fatigue performance decreased sharply. In this experiment, the number of fatigue cycles with an irregularity of 0 was three-fold higher than an irregularity of 0.5. In addition, when the irregularity was determined, the number of cycles also increased significantly with the decrease in porosity, which is consistent with the change of mechanical properties in the compression test. As shown in Figure 5B, the 4.1 MPa, 10.83 MPa and 23.43 MPa represent the stress values corresponding to the scaffold with porosity of 89%, 82% and 75% respectively, it can be seen that when the porosity is consistent, the greater the irregularity, the smaller the fatigue cycle number. Therefore, in order to ensure that the scaffold has excellent fatigue properties during structural design, it is necessary to control the irregularity within a reasonable range.

**FIGURE 6:**
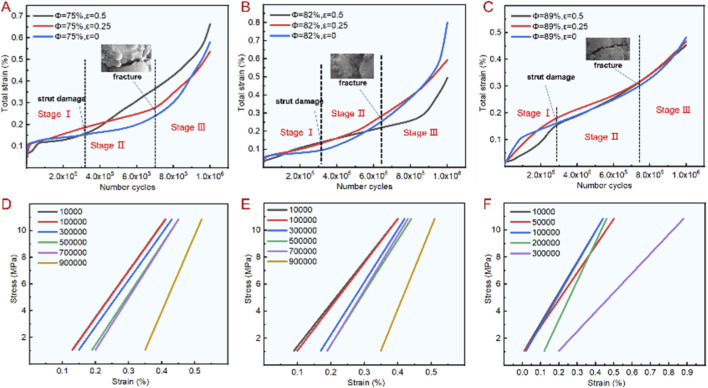
Fatigue test results of bionic porous structures. **(A–C)** Total fatigue strain with a designed porosity of 75%, 82 and 89. **(D–F)** The change trend of stress and strain corresponding to different cycles under the porosity of 82% and irregularity of 0, 0.25 and 0.5.

Based on the fatigue failure, the degree of deformation of the bionic porous structure was analyzed in various stages. The fatigue total strain process of the scaffold can be divided into three stages, as shown in Figures 6A–C. The first stage represents the cumulative process of plastic deformation caused by ratcheting effect. At this time, the scaffold does not break, and the curve changes relatively smoothly. In the second stage, fatigue fracture appeared and the total strain curve began to rise. The third stage indicates that the fatigue fracture increases rapidly until the scaffold is completely broken. It can also be seen that when the irregularity is 0.25, the fatigue strain generated is the largest, and when the irregularity is 0.5, the strain generated is the smallest, but its change rule is the same. With the increase of the number of cycles, the scaffold changes from strain accumulation to fatigue fracture, and the corresponding strain is larger when the number of cycles is maximum. Figures 6D–F show the change trend of stress and strain corresponding to different cycles under the porosity of 82% and irregularity of 0, 0.25 and 0.5. It can be seen that with the increase of cycles, the stress and strain curve is steeper, indicating that the corresponding elastic modulus is larger, especially when the fracture occurs in the third stage, the strain value is the largest.

According to the above fatigue characteristics analysis results, we further analysis the relationship between the number of fatigue cycles, porosity, and irregularity. Using the number of fatigue cycles as the dependent variable, and the irregularity and porosity as independent variables. We used IBM SPSS to process these data. Linear regression equation is used to evaluate the functional relationship between variables. Based on the number of fatigue cycles, irregularity, and porosity, and describe the mutual effects of these variables. The regression equation can be used to elucidate the effect of porosity and irregularity on the number of fatigue cycles. In addition, linear regression can not only research the relationship between independent variables and dependent variables, but also make more important mathematical predictions by establishing regression equations, reduce the experiments and provide analysis efficiency.

Statistical analysis confirmed that porosity and irregularity have a statistically significant effect on fatigue performance (p < 0.01). The determination coefficient *R* ^2^ = 0.901 indicates the regression equation exhibits excellent goodness-of-fit—90.1% of the variation in fatigue performance can be explained by porosity and irregularity—and the model has small prediction error. Linear regression Formula 4 is obtained from fatigue cycle number, irregularity, porosity and other parameters. Thus, compared with porosity, irregularity had a greater impact on the fatigue characteristics. When the proportion of loading force was similar, the fatigue performance showed a downward trend as the irregularity and porosity increased, which was also consistent with previous experimental results.

$$N=-4.42\times 10^{6}\times \emptyset -2.73\times 10^{6}\times \varepsilon +5.2\times 10^{6}$$ (4)

**FIGURE 7:**
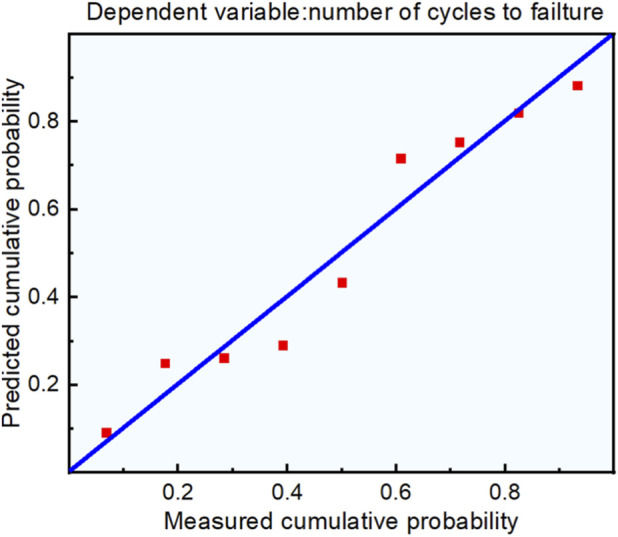
Normal P-P plot of standardized residuals.

We developed a linear regression equation based on two criteria: (1) Absence of multicollinearity between independent variables. (2) Normal distribution of the regression equation residuals. The residual normal P-P diagram of the regression equation is shown in Figure 7. The scattered points of the residuals basically were on or near the diagonal, suggesting normal distribution. The two conditions were satisfied, and therefore the linear regression equation was accurate and reliable.

Existing Voronoi-based porous scaffold studies mainly focused on quasi-static mechanical properties (e.g., compressive strength, elastic modulus) (Wang G. et al., 2018; Du et al., 2020; Liu et al., 2021). For example, Wang et al. (Wang G. et al., 2018) reported that Voronoi scaffolds with 70%–85% porosity have elastic moduli of 1.2–3.8 GPa, which is consistent with our results (1.414–4.281 GPa). However, these studies did not systematically investigate fatigue properties or their correlation with irregularity. Du et al. (Du et al., 2020) only analyzed the static compressive behavior of Voronoi scaffolds but ignored permeability and cell compatibility. In contrast, our study innovatively establishes the quantitative relationship between irregularity/porosity and fatigue performance (Equation 4), and comprehensively evaluates permeability and cell response—filling the gap in multi-performance integration design of Voronoi-based bone implants. Additionally, our fatigue cycle number (up to 23 × 10^5^ cycles for ε = 0, Φ = 75%) is significantly higher than the fatigue life of Voronoi scaffolds reported by Liu et al. (Liu et al., 2021) (≤10 × 10^5^ cycles), attributed to the parameterized control of pore irregularity.”

### 3.2 Permeability

**FIGURE 8:**
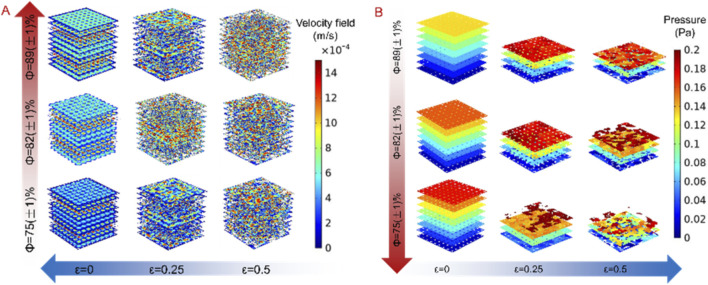
Fluid simulation results of bionic porous structures. **(A)** Velocity cloud diagram. **(B)** Pressure cloud diagram.

Permeability has a great impact on the biocompatibility of nutrient transport and bone tissue growth after bone implantation, which is an important parameter to determine the success rate of bone implantation (Shuai et al., 2020; Xie et al., 2023). Proper permeability is conducive to promoting the adhesion of cells and the formation of blood vessels on the bone implantation, and can greatly improve the depth of bone growth, but too high or too low permeability is not conducive to the application of bone implantation. When the porosity is too high, the mechanical properties will be reduced, the mechanical strength after bone implantation cannot be guaranteed. But when the porosity is too low, it will hinder the blood supply and bone tissue from growing to the depth. Therefore, when researching the permeability, it is necessary to comprehensively consider the mechanical and biological characteristics. In order to more intuitively analyze the flow characteristics under different design parameters, we analyze its flow rate and pressure distribution, as shown in Figure 8. From Figure 8A, we can see that the flow rate increases with the increase of porosity and decreases with the increase of irregularity, and the pressure distribution trend is opposite, as show in Figure 8B. According to the inverse relationship between flow velocity and pressure expressed in Formula 1, it can also be seen that although the distribution of flow velocity and pressure is disordered, but its flow direction has strong consistency, indicating that designed structure is conducive to guiding the flow in one direction.

**FIGURE 9:**
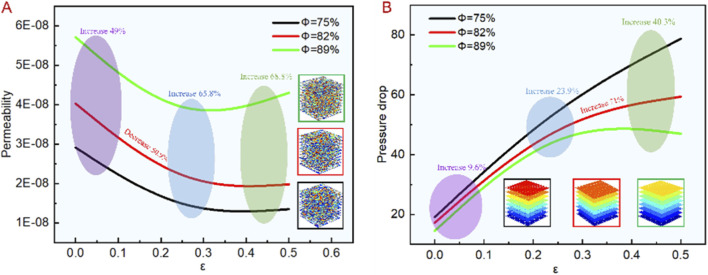
Fluid finite element analysis results of bionic porous structures. **(A)** Permeability change tend. **(B)** Pressure drop change tend.

In order to further analyze the flow characteristics of the designed model, we used Formulas 2, 3 to calculate the pressure drop and permeability with different parameters. The results are shown in Figure 9. From Figure 9A, it can be seen that under the same conditions, the permeability increases with the increase of porosity and decreases with the increase of irregularity, and the pressure drop is contrary to its change trend, which is consistent with the distribution trend of flow velocity cloud diagram. When the irregularity was 0, the permeability increased by 49% with the porosity increased. When the irregularity was 0.25, the permeability increased by 65.8% with the porosity increased. When the irregularity was 0.5, the permeability increases by 68.8% with the porosity increased. It can also be seen that the greater the irregularity, the greater the influence of porosity on permeability, because the smaller the irregularity is, the more regular the aperture distribution and the faster the flow velocity, and the permeability is relatively less affected by the porosity. When the irregularity increases, the aperture distribution is more chaotic, therefore, with the increased irregularity, the greater the porosity and permeability. Additionally, under constant porosity, the permeability decreases by 50.9% with the irregularity increased. Thus, the permeability can be controlled by adjusting the irregularity while the other conditions remain unchanged, and the control range is large. Figure 9B presents the effect of bionic porous structure on pressure drop under different irregularity and porosity, the pressure drop decrease with the porosity increased and increases with the irregularity increased, which is contrary to the change trend of permeability, and consistent with Formula 3. When the irregularity was 0 and 0.25, the pressure drop increased by 9.6% and 23.9% with the porosity increased. When the irregularity was 0.5, the pressure drop increased by 40.3% with the porosity increased. It can be seen that when the irregularity increases, the pressure drop is increasingly affected by the porosity, which is consistent with the effect on permeability. When the porosity is constant, the greater the irregularity, the greater the pressure drop. An increase of 71% indicates that the pressure drop can be controlled based on irregularity during structural design, with a large control range.

Some studies have shown that the permeability of natural bone is 0.4–11.0E-9 (Ali and Sen, 2017), while the permeability of bionic porous structure obtained by COMSOL simulation was 1.3–5.711E-8. The simulated permeability is 3–14 times higher than natural bone, which has dual biological implications: (1) Advantages: Higher permeability facilitates rapid nutrient transport (e.g., oxygen, growth factors) and metabolite excretion, promoting early cell adhesion and vascular ingrowth. (2) Risks: Excessively high permeability (e.g., ε=0 ; Φ=89% , permeability = 5.711E-8 m^2^) may reduce nutrient retention, leading to insufficient local nutrient concentration for cell proliferation. However, our study adjusts permeability to a moderate range (2.5-3.8E-8 m^2^) by controlling ε=0.25 , which balances nutrient transport and retention—explaining why ε=0.25 scaffolds show the best cell proliferation. Additionally, the higher permeability of the scaffold can be offset by the *in vivo* tissue ingrowth process: as bone tissue invades the pores, the effective permeability decreases to match natural bone, ensuring long-term osseointegration.

Thus, the permeability was higher than the level required for human bone. It can be seen from the simulation results that under the same condition, the influence of irregularity on the permeability is greater than porosity, so the design range of irregularity should be reasonably controlled during structural design.

**FIGURE 10:**
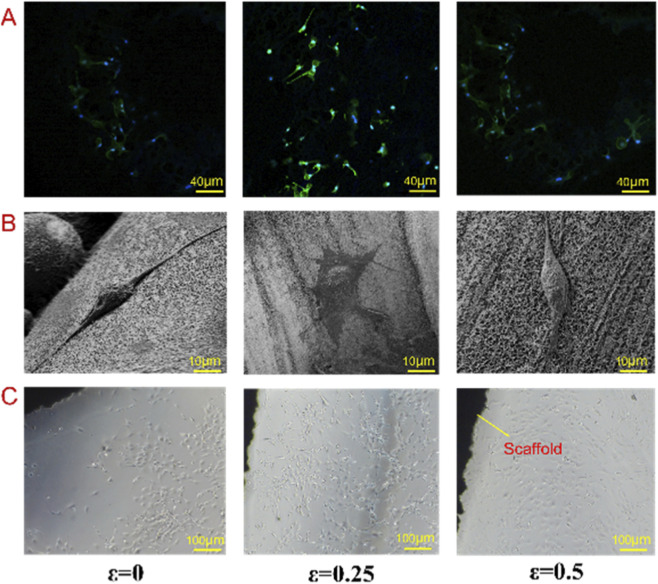
Cell test results. **(A)** Fluorescent staining results. **(B)** SEM analysis of porous structure. **(C)** Cell proliferation on the surface of each scaffold.

The above analysis shows that irregularity has a significant impact on permeability and fatigue characteristic, therefore, the bionic porous structure with porosity of 82% and corresponding irregularity of 0, 0.25 and 0.5 were selected for cell experiments. Figures 10A–C shows the fluorescent staining, the results of SEM morphology and cell proliferation on the surface of each scaffold. When the irregularity was 0, it can be seen from SEM images that the number of cells adhering to the scaffold surface is small and the extension of cell pseudopodium is small. And from the results of cell fluorescence staining and cell culture, it can be seen that there are relatively few connections between cells on the scaffold surface, and the order is relatively disordered. Indicating that high permeability is not conducive to cell adhesion and diffusion. When the irregularity increases to 0.25, the permeability decreases, from the SEM image, it can be seen that the pseudopodium of cells gradually extends, and from the results of fluorescence staining and cell culture, it can be seen that the number of cells also increases, and there is a strong directional arrangement along the pore direction. At the same time, the cells tend to cluster, which is conducive to the information transmission and gene expression between cells. However, when the irregularity is 0.5, the permeability of the scaffold reaches the lowest value. At this time, it can be seen from the SEM images that the number of cell pseudopods on the scaffold surface is less than that of the second group, and from the results of fluorescence staining and cell culture, the cells number of scaffold surface is reduced. Indicating that the low permeability is also not conducive to cell adhesion and inward growth. From the results of three groups cell culture, it can be seen that permeability has a great impact on the efficiency of cell inoculation, and the suitable permeability is conducive to the transfer of nutrients, metabolism, and the growth of bone tissue into the scaffold. Indicating that the resistance of cell culture medium to the scaffold is small, but when the permeability is too high or too low, the cell activity on the surface of the scaffold is not optimistic, and when the permeability is high, the resistance of cells passing through the scaffold is small, as a result, the cells stay on the surface of the scaffold for a short time, resulting in a small number of cells on the surface of the scaffold. When the permeability is too low, the resistance of cells passing through the scaffold is large, resulting in insufficient cell growth depth. Therefore, when selecting the permeability, it is necessary to control the permeability within an appropriate range to promote the number of cell proliferation and growth depth.

**FIGURE 11:**
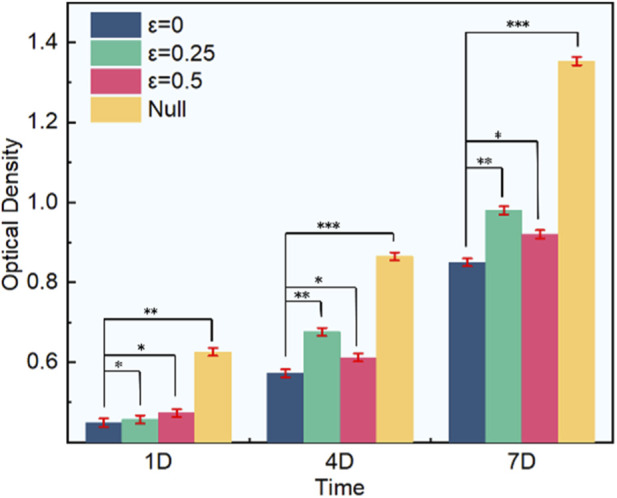
Proliferation of MC3T3-E1 cells on scaffolds: variation of OD value with irregularities.

In order to verify the influence of the above permeability on cell viability, we carried out OD test on three groups with different irregularity. The results are shown in Figure 11. On the first day of cell culture, the number of cells in each group was not significantly different. On the fourth day, the number of cells in each group increased significantly. When the irregularity was 0.25, the corresponding number of cells was the largest. On the seventh day, the number of cells in each group reached the maximum, and the number of cells with irregularity of 0.25 was greater than other two groups. The null group indicates that the scaffold is not placed, the cellular activity is higher, indicating that the scaffold affects the cell survival rate. The OD test results also show that the permeability has a great impact on the cell activity on the surface of the scaffold. Too high permeability easily leads to poor mechanical properties, and the number of cell adhesion on the surface of the scaffold is little, which is easy to lead to osteoporosis. On the contrary, when the permeability is low, it is not conducive to the nutrition transmission and bone tissue ingrowth after bone implantation, and it is easy to cause the poor combination between the bone implant and the tissue. Appropriate permeability is important for the depth of bone tissue growth while ensuring the mechanical properties.

## 4 Conclusion

This paper proposed a bionic porous structure design method using Voronoi principle. On this basis, the corresponding fatigue and permeability characteristics are evaluated, and also provided a functional relationship between fatigue characteristics and design parameters. Which beneficial to predict the fatigue characteristics of bone implant, and it is helpful to improve the theoretical value of bone implant in medical bioengineering. The main conclusions can be summarized as follows:

1. The Voronoi principle is introduced in the bionic porous scaffold design, the pore morphology and porosity of the scaffold can be controlled through parameters. The pore morphology is mainly controlled by adjusting the irregularity, and the porosity is controlled by adjusting the radius of the probability sphere. Therefore, achieving parameterized design of bionic porous scaffolds.
2. The fatigue test showed that the irregularity significantly affected the fatigue characteristics. When the porosity was determined, the fatigue characteristics decreased with the increase in irregularity, with the irregularity of 0 was three-fold higher than the irregularity of 0.5. Thus, bone implants can match the fatigue characteristics of natural bone by controlling the irregularity within a certain range.
3. The results of permeability experiment show that the irregularity has a great effect on cell adhesion and proliferation, and when other conditions remain unchanged, the permeability decreases with the increase in irregularity. And when the irregularity is 0.25, it is conducive to cell adhesion and proliferation.

## Data Availability

The original contributions presented in the study are included in the article/supplementary material, further inquiries can be directed to the corresponding authors.
